# Data-driven clustering approach to identify novel clusters of high cognitive impairment risk among Chinese community-dwelling elderly people with normal cognition: A national cohort study

**DOI:** 10.7189/jogh.14.04088

**Published:** 2024-04-19

**Authors:** Wang Ran, Qiutong Yu

**Affiliations:** 1 Zhejiang Provincial People’s Hospital, People’s Hospital of Hangzhou Medical College, Hangzhou, China; 2 Medical Education Department, Zhejiang Provincial People’s Hospital, People’s Hospital of Hangzhou Medical College, Hangzhou, China

## Abstract

**Background:**

Cognitive impairment is a highly heterogeneous disorder that necessitates further investigation into the distinct characteristics of populations at varying risk levels of cognitive impairment. Using a large-scale registry cohort of elderly individuals, we applied a data-driven approach to identify novel clusters based on diverse sociodemographic features.

**Methods:**

A prospective cohort of 6398 elderly people from the Chinese Longitudinal Healthy Longevity Survey, followed between 2008–14, was used to develop and validate the model. Participants were aged ≥60 years, community-dwelling, and the Chinese version of the Mini-Mental State Examination (MMSE) score ≥18 were included. Sixty-nine sociodemographic features were included in the analysis. The total population was divided into two-thirds for the derivation cohort (n = 4265) and one-third for the validation cohort (n = 2133). In the derivation cohort, an unsupervised Gaussian mixture model was applied to categorise participants into distinct clusters. A classifier was developed based on the most important 10 factors and was applied to categorise participants into their corresponding clusters in a validation cohort. The difference in the three-year risk of cognitive impairment was compared across the clusters.

**Results:**

We identified four clusters with distinct features in the derivation cohort. Cluster 1 was associated with the worst life independence, longest sleep duration, and the oldest age. Cluster 2 demonstrated the highest loneliness, characterised by non-marital status and living alone. Cluster 3 was characterised by the lowest sense of loneliness and the highest proportions in marital status and family co-residence. Cluster 4 demonstrated heightened engagement in exercise and leisure activity, along with independent decision-making, hygiene, and a diverse diet. In comparison to Cluster 4, Cluster 1 exhibited the highest three-year cognitive impairment risk (adjusted odds ratio (aOR) = 3.31; 95% confidence interval (CI) = 1.81–6.05), followed by Cluster 2 and Cluster 3 after adjustment for baseline MMSE, residence, sex, age, years of education, drinking, smoking, hypertension, diabetes, heart disease and stroke or cardiovascular diseases.

**Conclusions:**

A data-driven approach can be instrumental in identifying individuals at high risk of cognitive impairment among cognitively normal elderly populations. Based on various sociodemographic features, these clusters can suggest individualised intervention plans.

With the population ageing, cognitive impairment has become a pressing public health concern [1]. Research indicates a pervasive decline in cognitive function among older adults, with a doubling of the risk of dementia for every five-year increase in age for individuals aged >65 [2]. The prevalence of dementia exceeds 25% in those aged ≥90 [3]. Notably, China, with the largest ageing population globally, is undergoing rapid demographic ageing [4]. Various population-based studies have indicated a variable prevalence of mild cognitive impairment among Chinese older adults, ranging from 5–28% [5]. These findings underscore the growing threat posed by cognitive impairment.

Cognitive impairment is regarded as an intermediate stage between normal ageing and dementia, representing a considerably heterogeneous condition across diverse populations [6]. Despite recommendations for cognitive training, dietary interventions, physical exercise, social engagement, and other preventive measures against cognitive decline [7–9], some older adults still face a high risk of cognitive impairment. This underscores the need to identify further different subtypes that may lead to cognitive impairment, enabling more personalised interventions to prevent cognitive decline. Over the past two decades, cognitive impairment has evolved to accommodate heterogeneity in aetiologies and prognostic outcomes by differentiating it into various subtypes [6]. Recent studies indicate that, beyond relying solely on expert clinical knowledge, data-driven methods like unsupervised learning can unveil novel subtypes of various diseases, such as diabetes, hypertension, and other conditions [10,11].

The rapid expansion of the ageing population presents significant challenges to the widespread utilisation of subtype assessment based on serum markers. Therefore, it is imperative to utilise easily accessible features such as sociodemographic, health, and functional factors for a comprehensive, data-driven assessment of cognitive impairment heterogeneity. Standard statistical approaches, including regression analysis, emphasise the development of specific models and hypothesis testing to link predictors with a clinical diagnosis of cognitive impairment observed years later. However, these methods fall short of recognising population heterogeneity and identifying subgroups. Additionally, clinical diagnoses are prone to error during the earliest disease stages [12].

An alternative approach through unsupervised machine learning allows for the uncovering of inherent associations or clusters in unlabelled data independent of clinical diagnostic information, thus enabling an unbiased analysis [13,14]. Additionally, machine learning algorithms excel in processing and analysing data sets of high dimension and complexity, particularly sociodemographic factors [15]. Therefore, our study is focused on developing and evaluating novel clusters with differential risk factors for cognitive impairment among elderly individuals residing in Chinese communities with normal cognitive function, utilising unsupervised machine learning techniques. We included 69 features from the Chinese Longitudinal Healthy Longevity Survey (CLHLS), a nationally representative prospective cohort, to identify distinct clusters and provide individualised prevention strategies for cognitive impairment.

## METHODS

### Study design and population

**Figure 1:**
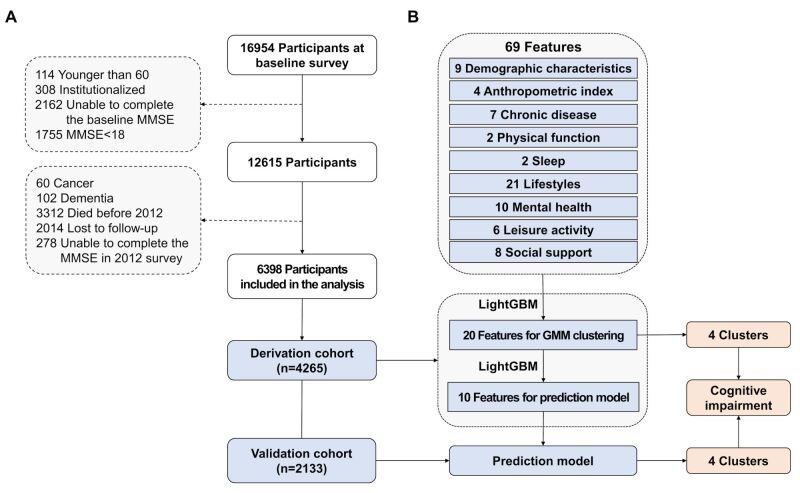
Study flowchart. **Panel A.** Patient selection. Two-thirds of the 6398 participants included in the analysis (n = 4265) were randomly assigned to the derivation cohort, while one-third (n = 2133) were randomly assigned to the validation cohort. **Panel B.** Study design. We included 69 features and identified the top 20 most important using LightGBM. Utilising these 20 features, the GMM was conducted in the derivation cohort to categorise participants into four clusters. The 10 most important features from the LightGBM model were selected to build another prediction model in the derivation cohort, which was applied to classify participants in the validation cohort. GMM – Gaussian Mixture Model, LightGBM – light gradient boosted machine

We included 6398 participants between 2008–12 in the national prospective longitudinal cohort CLHLS. Participants were included if they were 1) aged >60 years, 2) community-dwelling elderly people, and 3) had normal cognitive status (Mini-Mental State Examination (MMSE) score ≥18) in the survey of 2008–09 wave. They were excluded if they 1) were diagnosed with severe disease (cancer and dementia), 2) lived in an institution, or 3) there was a lack of follow-up cognitive assessment in the survey of the 2011–12 wave (**Figure 1**).

Further details about the CLHLS design and methodology have been described elsewhere. The survey results in the national database are freely accessible and available online [16]. A random split was applied to the included patients to divide them into two-thirds for the derivation cohort (n = 4265) and one-third for the validation cohort (n = 2133). The derivation cohort is where the model is built, and the validation cohort is where the model’s predictive validity and generalisability are tested (**Figure 1**).

Informed consent was obtained from all subjects involved in the study. Written informed consent was obtained from the patient(s) to publish this paper. The study was conducted in accordance with the Declaration of Helsinki and approved by the Ethical Review Committee of Peking University (IRB00001052-13074).

### Outcome variables

Cognitive impairment was defined by the Chinese version of the MMSE, which was culturally translated from the international standard of the MMSE questionnaire. The Chinese MMSE contains 24 items within six dimensions (five for orientation, three for registration, one for naming, five for attention and calculation, three for recall, and seven for language). The score of the Chinese MMSE ranges from 0–30 points, with higher scores indicating better cognition. The Chinese MMSE has been validated among the Chinese elderly population, and a score below 18 points in the survey of 2011–012 has been defined as cognitive impairment [17,18].

### Data pre-processing

We included 69 features related to cognitive impairment covering the domains of demographic characteristics (nine items), lifestyle (21 items), mental health (10 items), leisure activities (six items), sleep (two items), chronic diseases (seven items), physical function (two items), anthropometric index (four items) and social support (eight items) in the CLHLS database (Table S1 in the **Online Supplementary Document**). Data points in continuous variables exceeding four standard deviations were treated as missing values. The percentage of missing values for all variables was below 7% (Figure S1 in the **Online Supplementary Document**). The missing values were imputed with the mode of the data for categorical features and with the median of the data for numerical features. The participants were divided into eight subgroups according to their age (≤79, 80–89, 90–99, and ≥100) and gender (male and female), and the missing values were imputed based on the mode and the median of the respective subgroup.

### Feature selection

Within machine learning, ensemble learning is a general meta-approach that enhances predictive performance by combining the predictions from multiple models and typically exhibits superior generalisation capabilities compared to individual models [19]. As an ensemble algorithm, the light gradient boosted machine (LightGBM) demonstrates exceptional predictive accuracy and maintains stability in noise and outliers. Furthermore, it efficiently processes large-scale data sets [20].

To prioritise important features and eliminate irrelevant ones from the analysis, we employ LightGBM for feature selection within the derivation cohort. GridSearchCV was used for the grid search of hyperparameters, with the area under the receiver operating characteristics curve (AUC) serving as the evaluation metric. The information gain criteria and SHapley Additive exPlanations (SHAP) values were calculated to identify the importance of features.

### Unsupervised clustering analysis

To identify clusters of participants with similar features, clustering analysis was conducted on the data from the derivation cohort. Clustering analysis, a subset of unsupervised machine learning, was designed to categorise populations into multiple clusters based on similar features, ensuring high intra-cluster similarity and low inter-cluster similarity. The Gaussian Mixture Model (GMM) as a clustering approach was distinguished by its high generalisability and robustness. GMMs can adapt to a broad spectrum of data distributions encompassing tightly clustered, widely dispersed or overlapping data sets, offering exceptional modelling flexibility [21–23].

GMM constitutes a probabilistic model employing a soft clustering approach to group participants into discrete clusters, assuming that all data samples X are generated by a mixture of K multivariate Gaussian distributions. Here, each cluster is modelled as a Gaussian multivariate mixture with a mean and covariance that describes the shape of each cluster. In our analysis, the GMM model was trained using an iterative expectation-maximisation algorithm for 1000 epochs. Additionally, the optimal number of clusters for describing the derivation cohort data was determined using the Calinski Harabasz score [24]. Once the clusters were determined, patterns of features were visualised using an unsupervised hierarchical clustering heat map [25].

### Simplified supervised patient stratification model

To further reduce the dependence on multiple features in the stratification of participants, we selected the 10 most important features based on the information gain criteria from the LightGBM model, which was previously utilised for feature selection. Subsequently, an additional LightGBM prediction model based on the 10 most important features was developed using the data from the derivation cohort to classify participants into the corresponding cluster. The performance of the proposed prediction model in correctly assigning participants to clusters was assessed through a 10-fold cross-validation analysis utilising the AUC. Finally, the prediction model was applied to stratify the participants in the validation cohort. The clinical characteristics and outcomes in the sub-groups of the validation cohort were analysed to affirm the generalisability of the proposed clusters. For each cluster, radar plots were constructed based on the 10 key features, utilising z-values for each feature [26].

### Statistical analysis

Continuous variables were expressed as means and standard deviations (SDs). Categorical variables were expressed as frequencies and percentages. Univariate comparisons were conducted using the one-way ANOVA for continuous variables and the χ2 test for categorical data. Covariates known to be predictive of outcomes in cognitive impairment, such as gender, age, residential category (rural or urban), education level, smoking, drinking, hypertension, diabetes, and baseline MMSE total score, were adjusted in the multivariable models. Crude and multivariable-adjusted odds ratio (aOR) and 95% confidence interval (CI) for a three-year risk of cognitive impairment were obtained from a logistic regression model. All data were analysed with Python, version 3.7 (Python Software Foundation, Wilmington, DE, USA). The level of significance was defined as *P* < 0.05 (two-sided).

## RESULTS

### Population demographics

The study involved 6398 participants, characterised by an average age of 80 (SD = 10), 50.0% male, 2.76 years of education (SD = 3.73), 62.0% city/town, and 38.0% rural residents. The LightGBM model was developed using 69 features within the derivation cohort. Detailed information on all demographic features was presented in Tables S1–2 in the **Online Supplementary Document**.

### Feature selection

**Figure 2:**
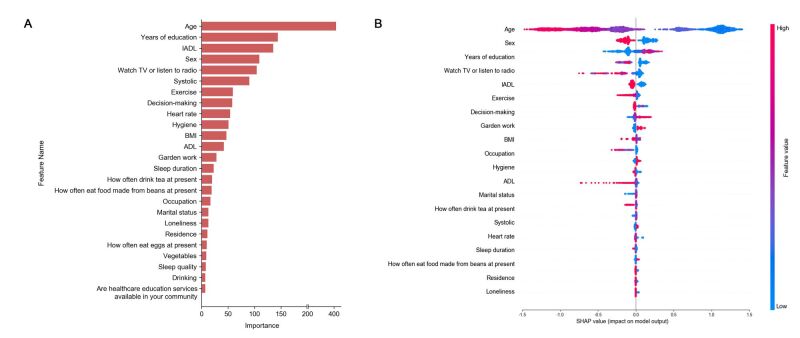
Importance ranking of features. **Panel A.** Importance ranking of features according to light gradient boosted machine model. The 25 most important features are depicted. **Panel B.** SHAP values for 20 features. ADL – activities of daily living, BMI – body mass index, IADL – instrumental activities of daily living, SHAP – SHapley Additive exPlanations

LightGBM was used to select features in the derivation cohort. The features were ranked according to their importance and evaluated by information gain criteria in the prediction of cognitive impairment. The 20 most important features were selected for further clustering analysis (**Figure 2**, panel A). SHapley Additive exPlanations values were calculated for these 20 features (**Figure 2**, panel B).

### Comparison of clinical characteristics among clusters

The GMM clustering method was employed to identify clusters of participants based on the 20 most important features. Based on the Calinski Harabasz score, four clusters were observed to be most optimal to represent the derivation cohort data (Figure S2 in the **Online Supplementary Document**). Thus, we identified four clusters with distinctive patterns of features, and the summary statistics of these clusters were presented in Table S1 in the **Online Supplementary Document**.

**Figure 3:**
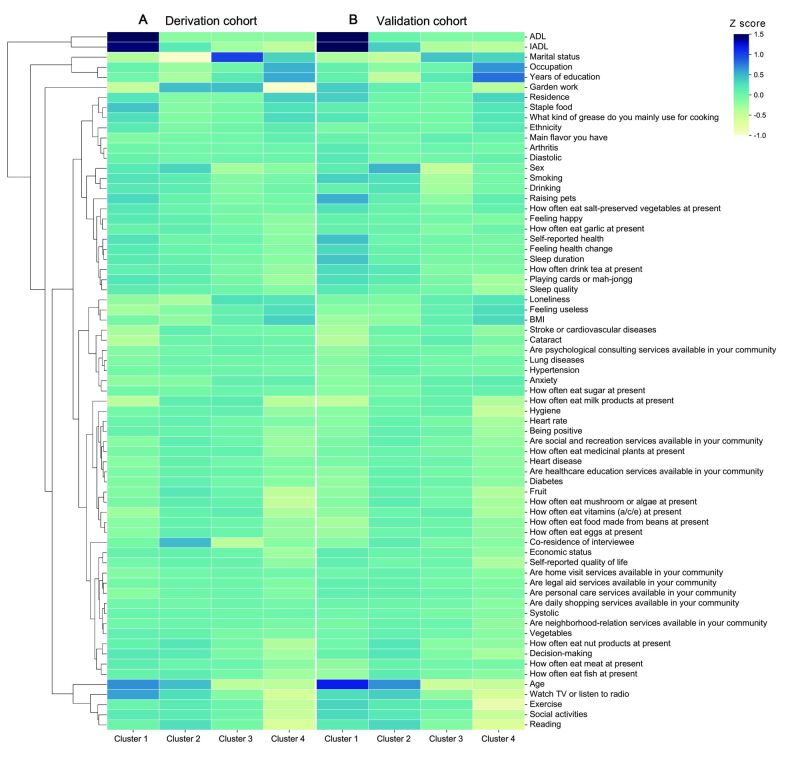
Dendrogram and heat map for unsupervised hierarchical clustering. Dendrogram and heat map for unsupervised hierarchical clustering in four clusters based on all the features. **Panel A.** Derivation cohort. **Panel B.** Validation cohort.

**Figure 3** illustrates the patterns of features among the four clusters. Post-analysis showed that Cluster 4, which included 678 (15.9%) participants, in comparison to the other three clusters, was characterised by heightened engagement in exercise and leisure activity, including playing cards or mah-jongg, reading, garden work, watching TV or listening to radio, and social activities, along with independent decision-making, hygiene, and a diverse diet with higher consumption of mushrooms, nuts, milk products, tea, eggs, fruits and plant-based oils. Cluster 3, including 1614 (37.8%) patients, had the lowest sense of loneliness and the highest proportions in marital status and family co-residence. In contrast, Cluster 2, including 1668 (39.1%) participants, demonstrates the highest sense of loneliness and is characterised by non-marital status and living alone. The 305 (7.2%) participants in Cluster 1 displayed the worst life independence, longest sleep duration, and oldest age (Table S1 in the **Online Supplementary Document**).

### Supervised prediction model

**Figure 4:**
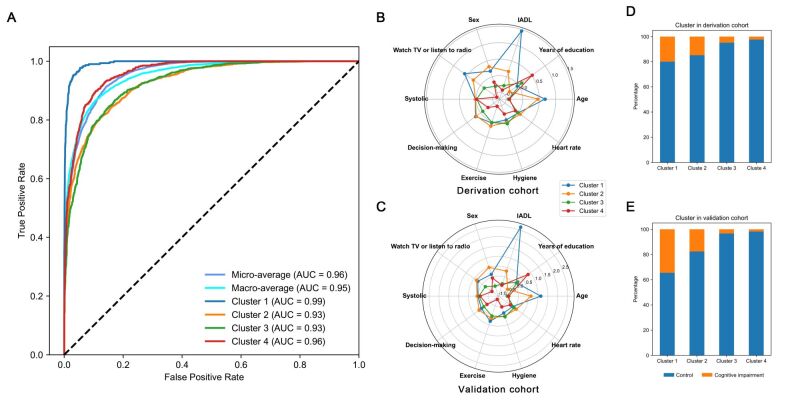
The predictive model accurately classifies the participants into their inherent clusters. **Panel A**. The receiver operating characteristic curves of prediction models. The performance of prediction models in assigning every participant to one of the four clusters. **Panels B–C**. The radar plots represent profiles of the four clusters identified in the derivation cohort (**B**) and validation cohort (**C**) based on 10 key features. Ten axes represented z-values for 10 key features. **Panels D–E**. The bar chart depicts the proportion of cognitive impairment at the three-year follow-up for each cluster in the derivation cohort (**D**) and validation cohort (**E**).

To further simplify the characterisation of the identified clusters, we selected the 10 most important features using the information gain criteria from the LightGBM model previously used for feature selection (**Figure 2**, panel A). Here, age, years of education, instrumental activities of daily living (IADL), sex, watching TV or listening to the radio, systole, exercise, decision-making, heart rate, and hygiene were observed to be the 10 most important features. Using these 10 model-derived, conventionally collected, important features, we developed a predictive model by the LightGBM to classify participants into one of the four clusters. In the 10-cross validation analysis on the derivation cohort data, the supervised prediction model achieved a four-class micro-average AUC of 0.955 (95% CI = 0.952–0.959) and a macro-average AUC of 0.952 (95% CI = 0.927–0.991). Cluster 1 AUC = 0.993; 95% CI = 0.990–0.995. Cluster 2 AUC = 0.928; 95% CI = 0.920–0.936. Cluster 3 AUC = 0.927; 95% CI = 0.919–0.934. Cluster 4 AUC = 0.962; 95% CI = 0.956–0.967 (**Figure 4**, panel A). Employing the same prediction model, participants from the validation cohort were allocated to one of the four clusters. The clusters in the validation cohort were observed to exhibit similar features as those in the derivation cohort (**Figure 3**, panel B). Radar plots were used to represent the profiles of the four clusters based on the 10 key features (**Figure 4**, panels B–C).

### Association of clusters with three-year cognitive impairment

The three-year risk of cognitive impairment across all the identified clusters was analysed. In the derivation cohort, Cluster 4 had the lowest three-year cognitive impairment rate (2.36%), followed by Cluster 3 (4.77%), Cluster 2 (14.93%), and Cluster 1 (20.00%) (**Figure 4**, panel D). In comparison to Cluster 4, Cluster 1 showed the highest three-year cognitive impairment risk (aOR = 3.31; 95% CI = 1.81–6.05, *P* < 0.001), followed by Cluster 2 (aOR = 2.36; 95% CI = 1.37–4.05, *P* = 0.002) and Cluster 3 (aOR = 1.88; 95% CI = 1.08–3.30, *P* = 0.027) after adjustment for baseline MMSE, residence, sex, age, years of education, drinking, smoking, hypertension, diabetes, heart disease and stroke or cardiovascular diseases (**Table 1**).

**Table 1:** The risk of three-year cognitive impairment*

Cohorts and clusters	Total population (n)	Cognitive impairment, n (%)	OR (95% CI)	Adjusted OR (95% CI)	*P*-value
Derivation cohort					
*Cluster 1*	305	61 (20.00)	10.34 (5.85–18.29)	3.31 (1.81–6.05)	<0.001
*Cluster 2*	1668	249 (14.93)	7.26 (4.34–12.14)	2.36 (1.37–4.05)	0.002
*Cluster 3*	1614	77 (4.77)	2.07 (1.20–3.58)	1.88 (1.08–3.30)	0.027
*Cluster 4*	678	16 (2.36)	–	–	–
Validation cohort					
*Cluster 1*	58	20 (34.48)	29.61 (9.59–91.39)	6.09 (1.82–20.37)	0.003
*Cluster 2*	904	159 (17.59)	12.01 (4.40–32.74)	3.11 (1.07–9.04)	0.037
*Cluster 3*	942	31 (3.29)	1.91 (0.67–5.48)	1.93 (0.64–5.77)	0.241
*Cluster 4*	229	4 (1.75)	–	–	–

CI – confidence interval, OR – odds ratio

*Adjusted for baseline Mini-Mental State Examination, residence, sex, age, years of education, drinking, smoking, hypertension, diabetes, heart disease and stroke or cardiovascular diseases.

A similar pattern was repeated in the validation cohort, and the participants in Cluster 4 were observed to have the lowest three-year cognitive impairment rate (1.75%), followed by Cluster 3 (3.29%), Cluster 2 (17.59%), and Cluster 1 (34.48%) (**Figure 4**, panel E). Clusters 1 and 2 exhibited a higher risk of cognitive impairment (Cluster 1 aOR = 6.09; 95% CI = 1.82–20.37, *P* = 0.003. Cluster 2 aOR = 3.11; 95% CI = 1.07–9.04, *P* = 0.037) at three-year follow-up compared to Cluster 4 (**Table 1**).

## DISCUSSION

In this prospective study, analysing 6398 elderly people from CLHLS register, we proposed a novel method for stratifying elderly individuals into four clusters, each with unique features and markedly varying risks of cognitive impairment. The proposed stratification approach for elderly individuals could provide insights into preventing cognitive decline. To the best of our knowledge, this is the first study that provides a novel stratification of elderly people based on 69 sociodemographic features, including demographic characteristics, anthropometric index, chronic disease, physical function, sleep, lifestyles, mental health, leisure activity and social support. Additionally, it uniquely applies machine learning techniques to address heterogeneity in cognitive impairment.

Despite numerous studies having developed predictive models for cognitive impairment, most have not fully utilised routinely collected demographic data, likely due to limitations inherent in traditional statistical methodologies [27]. For instance, the study by Walters et al., which utilised primary health care data and 14 clinical variables to predict dementia risk, demonstrated poor predictive performance in patients aged >80 [28]. Another study focusing on the primary health care population aged ≥75 achieved an AUC of 0.79 with fewer variables in a stepwise multivariate Cox proportional hazards model but had a complex evaluation process [29].

The most effective statistical technique for modelling with conventional demographic information has yet to be identified. Supervised machine learning has been employed to develop predictive models for revealing hidden dependencies within large data sets [30], such as Naïve Bayes, AdaBoost and Random Forest [31–33]. However, these approaches require labelled data for ‘diagnosis of cognitive impairment’ and exhibit variable external validity. Additionally, only about two-thirds of dementia patients are diagnosed, often at advanced stages of the disease [34]. The differentiation capability between cognitive impairment patients and healthy individuals is still insufficient. In our study, the unsupervised machine learning approach demonstrates superior flexibility, avoiding the need for labelled data, which is easier to collect and apply across various data sets [14].

There are few studies utilising unsupervised machine learning for cognitive impairment prediction. One study utilised hierarchical clustering on principal components to identify populations with a high likelihood of dementia in population-based surveys [35]. Another study investigated the longitudinal transition from normal to impaired functional status in an ageing survey population through unsupervised machine learning [36]. These studies employed a considerable number of variables for clustering. In contrast, our approach, through feature importance analysis and predictive models, enables the unique assignment of participants to the identified clusters using only 10 features. This ensures that the proposed method is applicable for extensive screening in remote areas.

Through unsupervised clustering analysis, we identified clusters with varying risks of cognitive impairment and demographic characteristics. This method is also efficient in studying risk factors of cognitive impairment and offers guidance for treatment plans. Diminished performance in IADL, reflecting the worst life independence, emerges as the predominant characteristic of Cluster 1, which is associated with the highest risk of cognitive impairment. IADL serves as a tool for assessing the ability of older adults to independently manage their daily lives, engage in social interactions, and accomplish household tasks [37]. IADL requires complex neuropsychological processing abilities, rendering it susceptible to impairment induced by cognitive decline [38,39]. Previous research has indicated that limitations in IADL can predict the transition to dementia over two [40] and four years [41]. Additionally, an unsupervised clustering study found that clusters at high risk of cognitive impairment had lower IADL scores and severe mobility impairments [35]. Our study also employed unsupervised clustering analysis, further underscoring the importance of IADL for identifying individuals at high risk of cognitive impairments. Consequently, the disability of the elderly cannot be ignored, and corresponding nursing and medical services, along with social support, should be taken promptly to delay cognitive decline.

Our study identified marital status and loneliness as critical factors for the identification of the elderly at high risk for cognitive impairment. The cluster associated with non-marital status and living alone was previously undetected in unsupervised clustering analyses. A growing body of literature indicates that social relationships are correlated with the incidence of dementia [42,43]. Marriage and cohabitation demonstrate a beneficial effect on dementia risk. A recent meta-analysis showed that elderly individuals who are lifelong singles or widowed have a 1.42-fold and 1.20-fold increased risk of dementia diagnosis, respectively, compared to their married counterparts [44]. The dissolution of marriage poses a significant threat to cognitive function and overall health in midlife, and remarriage can mitigate the cognitive declines associated with divorce [45]. Furthermore, a study involving 10 432 residents demonstrated that the cognitive detriment from widowhood in later life exceeds that of other risk factors such as smoking and drinking [46]. Another study established a supervised machine learning model to predict cognitive impairment, showing the highest accuracy in a model including marital status among four variables [47]. With the increasing prevalence of living alone and being unmarried among the elderly, this phenomenon merits further attention. The non-married elderly population requires more personalised care and social support to mitigate loneliness.

Within modifiable factors, our analysis revealed that individuals engaging more in exercise and leisure activity, such as garden work, watching TV or listening to the radio, alongside those adhering to a diverse diet (Cluster 4), exhibit the lowest risk of cognitive impairment. This finding aligns with extensive prior research indicating that regular exercise [48] and a healthy dietary pattern, incorporating items such as nuts, fruits, olive oil, mushrooms, tea, and dairy products, can reduce the risk of cognitive impairments in the elderly [49–54]. Additionally, the Finnish Geriatric Intervention Study to Prevent Cognitive Impairment and Disability (FINGER) study indicated that a comprehensive lifestyle intervention, encompassing mental health education, dietary guidance, physical exercise, cognitive training, and cardiovascular health management, can effectively prevent and slow the progression of cognitive decline, particularly in the elderly [8]. By identifying the characteristics of this cluster, we revealed the potential for reducing the risk of cognitive impairment by improving these factors. Offering platforms for leisure activities, fostering social interactions, promoting physical exercise, and providing volunteer services can, to some extent, help mitigate cognitive decline in the elderly community.

The results of this study have significant implications. First, using non-invasive, low-cost, and easy-to-acquire variables to develop a prediction model to stratify high-risk populations can reduce the health care costs of cognitive impairment screening. Second, this study incorporated a vast array of sociodemographic information, depicting comprehensive characteristics of the population to achieve better risk stratification, which could not have been possible with other methods focusing on only a few indicators. Third, in this analysis, the clusters were derived from a nationally representative prospective cohort, which reduced the ascertainment bias and underwent validation to ensure their generalisability. Furthermore, our study holds value for clinical practice by identifying individuals at a higher risk of cognitive impairments and elucidating their varying characteristics. With this information, health institutions can offer tailored preventative interventions to the elderly before any signs of cognitive deficits occur. Finally, this research has potential public health significance, providing effective and specific policy advice to policymakers for community-based elderly care in ageing countries such as China. Our finding is poised to facilitate the early detection, diagnosis, and treatment of cognitive impairments, potentially advancing public health management.

Our study has several limitations. First, during the follow-up process, a higher number of deaths and lost follow-ups, to some extent, reduced the statistical power. Second, machine learning outputs are inherently limited by inputs. Future research could incorporate routine biomarkers for a comprehensive analysis. Third, this study is based on participants from China, which may limit the extrapolation of the findings across different ethnicities. Further studies involving independent and ethnically diverse cohorts are necessary to generalise the findings of this study. Finally, some predictive factors used in our study were measured through self-reporting, introducing information bias. Despite these limitations, collecting self-reported data in primary health care settings is more feasible, and the results can be generalised to a wider community.

## CONCLUSIONS

Utilising data from a national cohort and employing machine learning techniques, we identified four distinct clusters associated with the three-year risk of cognitive impairment in the elderly population. Through a data-driven approach, this study proposes an effective stratification method for community-dwelling older adults, which is poised to play an important role in the screening and prevention of cognitive impairment.

## Additional material


Online Supplementary Document
