# Supplementary material for: Data-driven clustering approach to identify novel clusters of high cognitive impairment risk among Chinese community-dwelling elderly people with normal cognition: A national cohort study
Source: J Glob Health. 2024 Apr 19;14:04088. doi: 10.7189/jogh.14.04088 (PMC11026990; doi:10.7189/jogh.14.04088)
Supplement: Online Supplementary Document [file jogh-14-04088-s001.pdf]

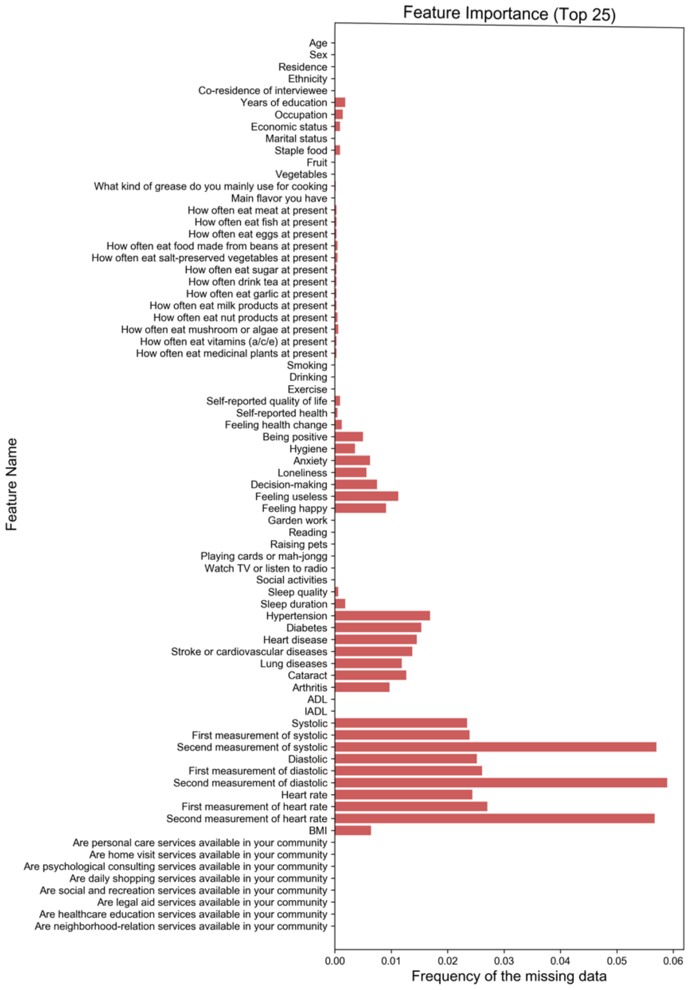

Figure S1. Frequency of the missing data.

Abbreviations: ADL, activities of daily living; IADL, instrumental activities of daily living; BMI, body mass index;

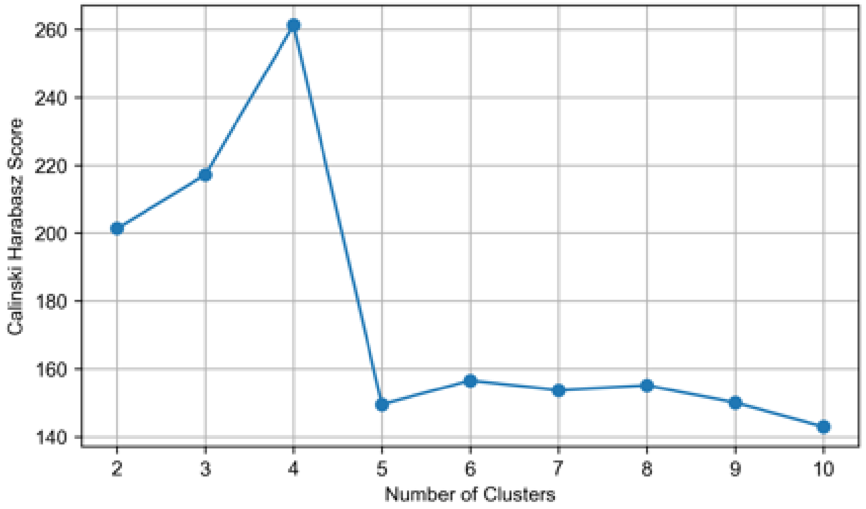

Figure S2. The identification of the optimal number of clusters.

Calinski harabasz (CH) score is a measure of the quality of the separation between clusters, for which higher values indicate more robust clustering.

Table S1. Cluster characteristics of the derivation cohort, stratified by gaussian mixture model.

**Features**		**Cluster 1 (N=305)**	**Cluster 2 (N=1668)**	**Cluster 3 (N=1614)**	**Cluster 4 (N=678)**	**P value**
**Demographic characteristics**						
Age #	—	86.52±9.90	84.31±9.56	75.43±8.40	75.67±8.10	<0.001
Sex #	Male	125 (40.98%)	564 (33.81%)	1049 (64.99%)	396 (58.41%)	<0.001
	Female	180 (59.02%)	1104 (66.19%)	565 (35.01%)	282 (41.59%)	
Residence #	Rural	157 (51.48%)	1136 (68.11%)	1074 (66.54%)	305 (44.99%)	<0.001
	City/town	148 (48.52%)	532 (31.89%)	540 (33.46%)	373 (55.01%)	
Ethnicity	Non-Han	9 (2.95%)	144 (8.63%)	106 (6.57%)	21 (3.10%)	<0.001
	Han	296 (97.05%)	1524 (91.37%)	1508 (93.43%)	657 (96.90%)	
Co-residence of interviewee	With household members	258 (84.59%)	1094 (65.59%)	1589 (98.45%)	607 (89.53%)	<0.001
	Alone	47 (15.41%)	574 (34.41%)	25 (1.55%)	71 (10.47%)	
Years of education #	—	2.68±4.08	1.60±2.85	3.31±3.76	4.80±4.58	<0.001
Occupation #	Agriculture/housework	214 (70.16%)	1390 (83.33%)	1171 (72.55%)	336 (49.56%)	<0.001
	Non-agriculture	91 (29.84%)	278 (16.67%)	443 (27.45%)	342 (50.44%)	
Economic status	Very rich	3 (0.98%)	15 (0.90%)	14 (0.87%)	13 (1.92%)	<0.001
	Rich	41 (13.44%)	191 (11.45%)	186 (11.52%)	142 (20.94%)	
	So so	205 (67.21%)	1142 (68.47%)	1148 (71.13%)	456 (67.26%)	
	Poor	44 (14.43%)	276 (16.55%)	232 (14.37%)	55 (8.11%)	
	Very poor	12 (3.93%)	44 (2.64%)	34 (2.11%)	12 (1.77%)	

Marital status #	No-married	204 (66.89%)	1668 (100.00%)	0 (0.00%)	219 (32.30%)	<0.001
	Married and living with spouse	101 (33.11%)	0 (0.00%)	1614 (100.00%)	459 (67.70%)	
**Anthropometric index**						
Systolic #	—	137.65±19.80	137.58±19.74	136.81±20.38	136.96±18.80	0.687
Diastolic	—	79.62±10.17	78.80±10.04	79.22±10.61	79.26±10.52	0.465
Heart rate #	—	73.65±7.88	74.25±8.16	73.45±8.28	72.73±8.53	<0.001
BMI #	—	20.79±3.60	20.29±3.33	21.32±3.30	22.19±3.61	<0.001
**Chronic disease**						
Hypertension	Yes	73 (23.93%)	368 (22.06%)	348 (21.56%)	166 (24.48%)	0.412
	No	232 (76.07%)	1300 (77.94%)	1266 (78.44%)	512 (75.52%)	
Diabetes	Yes	15 (4.92%)	24 (1.44%)	54 (3.35%)	38 (5.60%)	<0.001
	No	290 (95.08%)	1644 (98.56%)	1560 (96.65%)	640 (94.40%)	
Heart disease	Yes	43 (14.10%)	122 (7.31%)	159 (9.85%)	87 (12.83%)	<0.001
	No	262 (85.90%)	1546 (92.69%)	1455 (90.15%)	591 (87.17%)	
Stroke or cardiovascular diseases	Yes	36 (11.80%)	49 (2.94%)	88 (5.45%)	41 (6.05%)	<0.001
	No	269 (88.20%)	1619 (97.06%)	1526 (94.55%)	637 (93.95%)	
Lung diseases	Yes	39 (12.79%)	152 (9.11%)	158 (9.79%)	70 (10.32%)	0.244
	No	266 (87.21%)	1516 (90.89%)	1456 (90.21%)	608 (89.68%)	
Cataract	Yes	59 (19.34%)	138 (8.27%)	115 (7.13%)	57 (8.41%)	<0.001
	No	246 (80.66%)	1530 (91.73%)	1499 (92.87%)	621 (91.59%)	
Arthritis	Yes	69 (22.62%)	386 (23.14%)	371 (22.99%)	148 (21.83%)	0.917

	No	236 (77.38%)	1282 (76.86%)	1243 (77.01%)	530 (78.17%)	
**Physical function**						
ADL #	—	7.78±2.25	6.00±0.00	6.00±0.00	6.00±0.00	<0.001
IADL #	—	15.78±5.88	10.93±3.81	9.23±2.62	8.69±1.50	<0.001
**Sleep**						
	Very Good	40 (13.11%)	195 (11.69%)	235 (14.56%)	139 (20.50%)	
	Good	151 (49.51%)	890 (53.36%)	859 (53.22%)	345 (50.88%)	
Sleep quality	So so	68 (22.30%)	417 (25.00%)	352 (21.81%)	126 (18.58%)	<0.001
	Bad	41 (13.44%)	154 (9.23%)	155 (9.60%)	66 (9.73%)	
	Very bad	5 (1.64%)	12 (0.72%)	13 (0.81%)	2 (0.29%)	
Sleep duration #	—	8.05±2.45	7.85±2.10	7.63±1.86	7.41±1.85	<0.001
Smoking	Yes	42 (13.77%)	288 (17.27%)	458 (28.38%)	174 (25.66%)	<0.001
	No	263 (86.23%)	1380 (82.73%)	1156 (71.62%)	504 (74.34%)	
Drinking	Yes	43 (14.10%)	279 (16.73%)	427 (26.46%)	147 (21.68%)	<0.001
	No	262 (85.90%)	1389 (83.27%)	1187 (73.54%)	531 (78.32%)	
Exercise #	Yes	105 (34.43%)	492 (29.50%)	564 (34.94%)	406 (59.88%)	<0.001
	No	200 (65.57%)	1176 (70.50%)	1050 (65.06%)	272 (40.12%)	
**Lifestyles**						
	Rice	120 (39.34%)	1148 (68.82%)	1028 (63.69%)	341 (50.29%)	
Staple food	Corn (maize)	19 (6.23%)	42 (2.52%)	45 (2.79%)	26 (3.83%)	<0.001
	Wheat (noodles and bread etc.)	100 (32.79%)	284 (17.03%)	357 (22.12%)	168 (24.78%)	

	Rice and wheat	66 (21.64%)	194 (11.63%)	184 (11.40%)	143 (21.09%)	
Fruit	Almost everyday	64 (20.98%)	139 (8.33%)	173 (10.72%)	174 (25.66%)	<0.001
	Quite often	77 (25.25%)	403 (24.16%)	453 (28.07%)	234 (34.51%)	
	Occasionally	93 (30.49%)	690 (41.37%)	652 (40.40%)	189 (27.88%)	
	Rarely or never	71 (23.28%)	436 (26.14%)	336 (20.82%)	81 (11.95%)	
Vegetables	Almost everyday	188 (61.64%)	1078 (64.63%)	1131 (70.07%)	482 (71.09%)	<0.001
	Quite often	90 (29.51%)	419 (25.12%)	367 (22.74%)	155 (22.86%)	
	Occasionally	23 (7.54%)	147 (8.81%)	104 (6.44%)	34 (5.01%)	
	Rarely or never	4 (1.31%)	24 (1.44%)	12 (0.74%)	7 (1.03%)	
What kind of grease do you mainly use for cooking	Animal fat	18 (5.90%)	334 (20.02%)	275 (17.04%)	36 (5.31%)	<0.001
	Plant oil	287 (94.10%)	1334 (79.98%)	1339 (82.96%)	642 (94.69%)	
Main flavor you have	No-salty	263 (86.23%)	1378 (82.61%)	1331 (82.47%)	527 (77.73%)	<0.001
	Salty	42 (13.77%)	290 (17.39%)	283 (17.53%)	151 (22.27%)	
How often eat meat at present	Almost everyday	75 (24.59%)	484 (29.02%)	447 (27.70%)	252 (37.17%)	<0.001
	Once a week	121 (39.67%)	620 (37.17%)	638 (39.53%)	266 (39.23%)	
	Once a month	33 (10.82%)	217 (13.01%)	211 (13.07%)	52 (7.67%)	
	Sometimes	41 (13.44%)	197 (11.81%)	166 (10.29%)	57 (8.41%)	
	Never	35 (11.48%)	150 (8.99%)	152 (9.42%)	51 (7.52%)	
How often eat fish at present	Almost everyday	26 (8.52%)	90 (5.40%)	125 (7.74%)	73 (10.77%)	<0.001
	Once a week	92 (30.16%)	494 (29.62%)	523 (32.40%)	291 (42.92%)	
	Once a month	67 (21.97%)	342 (20.50%)	364 (22.55%)	128 (18.88%)	

	Sometimes	39 (12.79%)	333 (19.96%)	300 (18.59%)	92 (13.57%)	
	Never	81 (26.56%)	409 (24.52%)	302 (18.71%)	94 (13.86%)	
How often eat eggs at present	Almost everyday	138 (45.25%)	477 (28.60%)	497 (30.79%)	310 (45.72%)	<0.001
	Once a week	91 (29.84%)	602 (36.09%)	604 (37.42%)	226 (33.33%)	
	Once a month	24 (7.87%)	235 (14.09%)	217 (13.44%)	50 (7.37%)	
	Sometimes	23 (7.54%)	173 (10.37%)	151 (9.36%)	40 (5.90%)	
	Never	29 (9.51%)	181 (10.85%)	145 (8.98%)	52 (7.67%)	
How often eat food made from beans at present #	Almost everyday	81 (26.56%)	255 (15.29%)	255 (15.80%)	169 (24.93%)	<0.001
	Once a week	107 (35.08%)	621 (37.23%)	616 (38.17%)	272 (40.12%)	
	Once a month	40 (13.11%)	263 (15.77%)	253 (15.68%)	92 (13.57%)	
	Sometimes	43 (14.10%)	314 (18.82%)	297 (18.40%)	88 (12.98%)	
	Never	34 (11.15%)	215 (12.89%)	193 (11.96%)	57 (8.41%)	
How often eat salt-preserved vegetables at present	Almost everyday	56 (18.36%)	348 (20.86%)	391 (24.23%)	163 (24.04%)	<0.001
	Once a week	50 (16.39%)	288 (17.27%)	297 (18.40%)	136 (20.06%)	
	Once a month	20 (6.56%)	164 (9.83%)	156 (9.67%)	56 (8.26%)	
	Sometimes	40 (13.11%)	270 (16.19%)	260 (16.11%)	102 (15.04%)	
	Never	139 (45.57%)	598 (35.85%)	510 (31.60%)	221 (32.60%)	
How often eat sugar at present	Almost everyday	52 (17.05%)	243 (14.57%)	181 (11.21%)	97 (14.31%)	<0.001
	Once a week	59 (19.34%)	315 (18.88%)	331 (20.51%)	133 (19.62%)	
	Once a month	27 (8.85%)	217 (13.01%)	174 (10.78%)	91 (13.42%)	
	Sometimes	49 (16.07%)	384 (23.02%)	397 (24.60%)	125 (18.44%)	

	Never	118 (38.69%)	509 (30.52%)	531 (32.90%)	232 (34.22%)	
How often drink tea at present #	Almost everyday	94 (30.82%)	519 (31.12%)	612 (37.92%)	315 (46.46%)	<0.001
	Once a week	20 (6.56%)	83 (4.98%)	104 (6.44%)	38 (5.60%)	
	Once a month	7 (2.30%)	48 (2.88%)	43 (2.66%)	15 (2.21%)	
	Sometimes	27 (8.85%)	121 (7.25%)	133 (8.24%)	42 (6.19%)	
	Never	157 (51.48%)	897 (53.78%)	722 (44.73%)	268 (39.53%)	
How often eat garlic at present	Almost everyday	63 (20.66%)	329 (19.72%)	369 (22.86%)	205 (30.24%)	<0.001
	Once a week	71 (23.28%)	337 (20.20%)	363 (22.49%)	161 (23.75%)	
	Once a month	34 (11.15%)	178 (10.67%)	174 (10.78%)	70 (10.32%)	
	Sometimes	54 (17.70%)	397 (23.80%)	375 (23.23%)	108 (15.93%)	
	Never	83 (27.21%)	427 (25.60%)	333 (20.63%)	134 (19.76%)	
How often eat milk products at present	Almost everyday	89 (29.18%)	221 (13.25%)	195 (12.08%)	198 (29.20%)	<0.001
	Once a week	45 (14.75%)	140 (8.39%)	139 (8.61%)	104 (15.34%)	
	Once a month	20 (6.56%)	104 (6.24%)	106 (6.57%)	31 (4.57%)	
	Sometimes	33 (10.82%)	283 (16.97%)	241 (14.93%)	91 (13.42%)	
	Never	118 (38.69%)	920 (55.16%)	933 (57.81%)	254 (37.46%)	
How often eat nut products at present	Almost everyday	12 (3.93%)	41 (2.46%)	73 (4.52%)	52 (7.67%)	<0.001
	Once a week	25 (8.20%)	112 (6.71%)	160 (9.91%)	119 (17.55%)	
	Once a month	31 (10.16%)	121 (7.25%)	164 (10.16%)	79 (11.65%)	
	Sometimes	58 (19.02%)	344 (20.62%)	439 (27.20%)	160 (23.60%)	
	Never	179 (58.69%)	1050 (62.95%)	778 (48.20%)	268 (39.53%)	

How often eat mushroom or algae at present	Almost everyday	6 (1.97%)	16 (0.96%)	25 (1.55%)	38 (5.60%)	<0.001
	Once a week	46 (15.08%)	131 (7.85%)	129 (7.99%)	153 (22.57%)	
	Once a month	39 (12.79%)	195 (11.69%)	230 (14.25%)	124 (18.29%)	
	Sometimes	82 (26.89%)	544 (32.61%)	514 (31.85%)	164 (24.19%)	
	Never	132 (43.28%)	782 (46.88%)	716 (44.36%)	199 (29.35%)	
How often eat vitamins (a/c/e) at present	Almost everyday	35 (11.48%)	72 (4.32%)	63 (3.90%)	85 (12.54%)	<0.001
	Once a week	16 (5.25%)	45 (2.70%)	41 (2.54%)	43 (6.34%)	
	Once a month	14 (4.59%)	54 (3.24%)	42 (2.60%)	23 (3.39%)	
	Sometimes	38 (12.46%)	222 (13.31%)	184 (11.40%)	94 (13.86%)	
	Never	202 (66.23%)	1275 (76.44%)	1284 (79.55%)	433 (63.86%)	
How often eat medicinal plants at present	Almost everyday	6 (1.97%)	8 (0.48%)	17 (1.05%)	23 (3.39%)	<0.001
	Once a week	6 (1.97%)	21 (1.26%)	23 (1.43%)	31 (4.57%)	
	Once a month	15 (4.92%)	44 (2.64%)	40 (2.48%)	21 (3.10%)	
	Sometimes	32 (10.49%)	216 (12.95%)	172 (10.66%)	81 (11.95%)	
	Never	246 (80.66%)	1379 (82.67%)	1362 (84.39%)	522 (76.99%)	
**Mental health**						
Self-reported quality of life	Very good	54 (17.70%)	184 (11.03%)	209 (12.95%)	145 (21.39%)	<0.001
	Good	134 (43.93%)	809 (48.50%)	709 (43.93%)	306 (45.13%)	
	So so	95 (31.15%)	553 (33.15%)	601 (37.24%)	203 (29.94%)	
	Bad	17 (5.57%)	109 (6.53%)	86 (5.33%)	23 (3.39%)	
	Very bad	5 (1.64%)	13 (0.78%)	9 (0.56%)	1 (0.15%)	

Self-reported health	Very good	38 (12.46%)	205 (12.29%)	214 (13.26%)	132 (19.47%)	<0.001
	Good	103 (33.77%)	703 (42.15%)	658 (40.77%)	261 (38.50%)	
	So so	94 (30.82%)	554 (33.21%)	518 (32.09%)	230 (33.92%)	
	Bad	60 (19.67%)	194 (11.63%)	208 (12.89%)	50 (7.37%)	
	Very bad	10 (3.28%)	12 (0.72%)	16 (0.99%)	5 (0.74%)	
Feeling health change	Much better	10 (3.28%)	27 (1.62%)	40 (2.48%)	22 (3.24%)	<0.001
	A little better	23 (7.54%)	137 (8.21%)	157 (9.73%)	72 (10.62%)	
	No change	141 (46.23%)	947 (56.77%)	910 (56.38%)	400 (59.00%)	
	A little worse	111 (36.39%)	504 (30.22%)	446 (27.63%)	163 (24.04%)	
	Much worse	20 (6.56%)	53 (3.18%)	61 (3.78%)	21 (3.10%)	
Being positive	Always	49 (16.07%)	185 (11.09%)	215 (13.32%)	160 (23.60%)	<0.001
	Often	193 (63.28%)	1152 (69.06%)	1104 (68.40%)	421 (62.09%)	
	Sometimes	41 (13.44%)	268 (16.07%)	233 (14.44%)	80 (11.80%)	
	Seldom	18 (5.90%)	55 (3.30%)	59 (3.66%)	11 (1.62%)	
	Never	4 (1.31%)	8 (0.48%)	3 (0.19%)	6 (0.88%)	
Hygiene #	Always	46 (15.08%)	190 (11.39%)	197 (12.21%)	161 (23.75%)	<0.001
	Often	187 (61.31%)	1069 (64.09%)	970 (60.10%)	380 (56.05%)	
	Sometimes	68 (22.30%)	384 (23.02%)	414 (25.65%)	131 (19.32%)	
	Seldom	4 (1.31%)	22 (1.32%)	30 (1.86%)	5 (0.74%)	
	Never	0 (0.00%)	3 (0.18%)	3 (0.19%)	1 (0.15%)	
Anxiety	Always	4 (1.31%)	29 (1.74%)	15 (0.93%)	20 (2.95%)	<0.001

	Often	21 (6.89%)	71 (4.26%)	52 (3.22%)	20 (2.95%)	
	Sometimes	77 (25.25%)	372 (22.30%)	279 (17.29%)	98 (14.45%)	
	Seldom	95 (31.15%)	605 (36.27%)	539 (33.40%)	214 (31.56%)	
	Never	108 (35.41%)	591 (35.43%)	729 (45.17%)	326 (48.08%)	
Loneliness #	Always	7 (2.30%)	45 (2.70%)	9 (0.56%)	11 (1.62%)	<0.001
	Often	23 (7.54%)	130 (7.79%)	30 (1.86%)	25 (3.69%)	
	Sometimes	73 (23.93%)	469 (28.12%)	200 (12.39%)	88 (12.98%)	
	Seldom	93 (30.49%)	517 (31.00%)	526 (32.59%)	171 (25.22%)	
	Never	109 (35.74%)	507 (30.40%)	849 (52.60%)	383 (56.49%)	
Decision-making #	Always	96 (31.48%)	556 (33.33%)	638 (39.53%)	340 (50.15%)	<0.001
	Often	77 (25.25%)	408 (24.46%)	493 (30.55%)	181 (26.70%)	
	Sometimes	76 (24.92%)	407 (24.40%)	323 (20.01%)	114 (16.81%)	
	Seldom	44 (14.43%)	224 (13.43%)	104 (6.44%)	29 (4.28%)	
	Never	12 (3.93%)	73 (4.38%)	56 (3.47%)	14 (2.06%)	
Feeling useless	Always	33 (10.82%)	126 (7.55%)	74 (4.58%)	28 (4.13%)	<0.001
	Often	66 (21.64%)	303 (18.17%)	237 (14.68%)	73 (10.77%)	
	Sometimes	107 (35.08%)	605 (36.27%)	558 (34.57%)	210 (30.97%)	
	Seldom	61 (20.00%)	353 (21.16%)	370 (22.92%)	153 (22.57%)	
	Never	38 (12.46%)	281 (16.85%)	375 (23.23%)	214 (31.56%)	
Feeling happy	Always	25 (8.20%)	121 (7.25%)	114 (7.06%)	81 (11.95%)	<0.001
	Often	44 (14.43%)	303 (18.17%)	326 (20.20%)	129 (19.03%)	

	Sometimes	96 (31.48%)	508 (30.46%)	620 (38.41%)	268 (39.53%)	
	Seldom	88 (28.85%)	534 (32.01%)	422 (26.15%)	151 (22.27%)	
	Never	52 (17.05%)	202 (12.11%)	132 (8.18%)	49 (7.23%)	
**Leisure activity**						
Garden work #	Almost everyday	74 (24.26%)	0 (0.00%)	0 (0.00%)	452 (66.67%)	<0.001
	Once a week	16 (5.25%)	0 (0.00%)	0 (0.00%)	89 (13.13%)	
	Once a month	10 (3.28%)	0 (0.00%)	0 (0.00%)	54 (7.96%)	
	Sometimes	30 (9.84%)	0 (0.00%)	0 (0.00%)	83 (12.24%)	
	Never	175 (57.38%)	1668 (100.00%)	1614 (100.00%)	0 (0.00%)	
Reading	Almost everyday	41 (13.44%)	102 (6.12%)	219 (13.57%)	204 (30.09%)	<0.001
	Once a week	7 (2.30%)	36 (2.16%)	77 (4.77%)	60 (8.85%)	
	Once a month	10 (3.28%)	25 (1.50%)	47 (2.91%)	32 (4.72%)	
	Sometimes	16 (5.25%)	35 (2.10%)	80 (4.96%)	51 (7.52%)	
	Never	231 (75.74%)	1470 (88.13%)	1191 (73.79%)	331 (48.82%)	
Raising pets	Almost everyday	44 (14.43%)	388 (23.26%)	480 (29.74%)	176 (25.96%)	<0.001
	Once a week	5 (1.64%)	58 (3.48%)	63 (3.90%)	28 (4.13%)	
	Once a month	4 (1.31%)	22 (1.32%)	25 (1.55%)	21 (3.10%)	
	Sometimes	8 (2.62%)	52 (3.12%)	46 (2.85%)	21 (3.10%)	
	Never	244 (80.00%)	1148 (68.82%)	1000 (61.96%)	432 (63.72%)	
Playing cards or mah-jongg	Almost everyday	12 (3.93%)	101 (6.06%)	144 (8.92%)	85 (12.54%)	<0.001
	Once a week	11 (3.61%)	86 (5.16%)	105 (6.51%)	59 (8.70%)	

	Once a month	5 (1.64%)	43 (2.58%)	54 (3.35%)	39 (5.75%)	
	Sometimes	13 (4.26%)	46 (2.76%)	86 (5.33%)	42 (6.19%)	
	Never	264 (86.56%)	1392 (83.45%)	1225 (75.90%)	453 (66.81%)	
Watch TV or listen to radio #	Almost everyday	116 (38.03%)	856 (51.32%)	1124 (69.64%)	634 (93.51%)	<0.001
	Once a week	31 (10.16%)	203 (12.17%)	172 (10.66%)	44 (6.49%)	
	Once a month	28 (9.18%)	101 (6.06%)	68 (4.21%)	0 (0.00%)	
	Sometimes	32 (10.49%)	118 (7.07%)	65 (4.03%)	0 (0.00%)	
	Never	98 (32.13%)	390 (23.38%)	185 (11.46%)	0 (0.00%)	
Social activities	Almost everyday	7 (2.30%)	36 (2.16%)	55 (3.41%)	65 (9.59%)	<0.001
	Once a week	6 (1.97%)	28 (1.68%)	40 (2.48%)	32 (4.72%)	
	Once a month	6 (1.97%)	41 (2.46%)	58 (3.59%)	58 (8.55%)	
	Sometimes	9 (2.95%)	72 (4.32%)	113 (7.00%)	99 (14.60%)	
	Never	277 (90.82%)	1491 (89.39%)	1348 (83.52%)	424 (62.54%)	
**Social support**						
Are personal care services available in your community	Yes	19 (6.23%)	55 (3.30%)	43 (2.66%)	36 (5.31%)	<0.001
	No	286 (93.77%)	1613 (96.70%)	1571 (97.34%)	642 (94.69%)	
Are home visit services available in your community	Yes	31 (10.16%)	117 (7.01%)	101 (6.26%)	49 (7.23%)	0.106
	No	274 (89.84%)	1551 (92.99%)	1513 (93.74%)	629 (92.77%)	
Are psychological consulting services available in your community	Yes	21 (6.89%)	77 (4.62%)	53 (3.28%)	28 (4.13%)	0.023
	No	284 (93.11%)	1591 (95.38%)	1561 (96.72%)	650 (95.87%)	
	Yes	13 (4.26%)	67 (4.02%)	54 (3.35%)	29 (4.28%)	0.634

Are daily shopping services available in your community	No	292 (95.74%)	1601 (95.98%)	1560 (96.65%)	649 (95.72%)	
Are social and recreation services available in your community	Yes	43 (14.10%)	107 (6.41%)	146 (9.05%)	113 (16.67%)	<0.001
	No	262 (85.90%)	1561 (93.59%)	1468 (90.95%)	565 (83.33%)	
Are legal aid services available in your community	Yes	20 (6.56%)	84 (5.04%)	89 (5.51%)	56 (8.26%)	0.021
	No	285 (93.44%)	1584 (94.96%)	1525 (94.49%)	622 (91.74%)	
Are healthcare education services available in your community	Yes	29 (9.51%)	98 (5.88%)	98 (6.07%)	72 (10.62%)	<0.001
	No	276 (90.49%)	1570 (94.12%)	1516 (93.93%)	606 (89.38%)	
Are neighborhood-relation services available in your community	Yes	51 (16.72%)	262 (15.71%)	262 (16.23%)	127 (18.73%)	0.347
	No	254 (83.28%)	1406 (84.29%)	1352 (83.77%)	551 (81.27%)	

[#]The 20 features used in the clustering analysis. Abbreviations: ADL, activities of daily living; IADL, instrumental activities of daily living; BMI, body mass index;

Table S2. Cluster characteristics of the validation cohort, stratified by gaussian mixture model.

Features		Cluster 1 (N=58)	Cluster 2 (N=904)	Cluster 3 (N=942)	Cluster 4 (N=229)	P value
**Demographic characteristics**						
Age #	—	91.47±10.30	86.38±8.67	74.22±7.43	74.92±7.08	<0.001
Sex #	Male	25 (43.10%)	221 (24.45%)	701 (74.42%)	121 (52.84%)	<0.001
	Female	33 (56.90%)	683 (75.55%)	241 (25.58%)	108 (47.16%)	
Residence #	Rural	26 (44.83%)	561 (62.06%)	602 (63.91%)	106 (46.29%)	<0.001
	City/town	32 (55.17%)	343 (37.94%)	340 (36.09%)	123 (53.71%)	
Ethnicity	Non-Han	5 (8.62%)	57 (6.31%)	68 (7.22%)	6 (2.62%)	0.071
	Han	53 (91.38%)	847 (93.69%)	874 (92.78%)	223 (97.38%)	
Co-residence of interviewee	With household members	51 (87.93%)	708 (78.32%)	813 (86.31%)	204 (89.08%)	<0.001
	Alone	7 (12.07%)	196 (21.68%)	129 (13.69%)	25 (10.92%)	
Years of education #	—	3.05±4.71	1.04±2.43	3.34±3.57	5.68±4.36	<0.001
Occupation #	Agriculture/housework	40 (68.97%)	726 (80.31%)	673 (71.44%)	101 (44.10%)	<0.001
	Non-agriculture	18 (31.03%)	178 (19.69%)	269 (28.56%)	128 (55.90%)	
Economic status	Very rich	1 (1.72%)	12 (1.33%)	8 (0.85%)	5 (2.18%)	<0.001
	Rich	6 (10.34%)	118 (13.05%)	102 (10.83%)	39 (17.03%)	
	So so	38 (65.52%)	609 (67.37%)	697 (73.99%)	160 (69.87%)	
	Poor	11 (18.97%)	133 (14.71%)	119 (12.63%)	24 (10.48%)	
	Very poor	2 (3.45%)	32 (3.54%)	16 (1.70%)	1 (0.44%)	

Marital status #	No-married	38 (65.52%)	652 (72.12%)	271 (28.77%)	75 (32.75%)	<0.001
	Married and living with spouse	20 (34.48%)	252 (27.88%)	671 (71.23%)	154 (67.25%)	
**Anthropometric index**						
Systolic #	—	136.49±18.11	137.35±19.77	135.53±19.63	134.34±19.68	0.101
Diastolic	—	80.93±12.29	78.30±10.48	78.88±10.54	79.57±10.54	0.133
Heart rate #	—	72.20±6.81	74.11±8.02	72.98±8.32	71.02±8.81	<0.001
BMI #	—	20.14±4.11	20.32±3.53	21.34±3.23	22.01±3.47	<0.001
**Chronic disease**						
Hypertension	Yes	17 (29.31%)	177 (19.58%)	212 (22.51%)	58 (25.33%)	0.090
	No	41 (70.69%)	727 (80.42%)	730 (77.49%)	171 (74.67%)	
Diabetes	Yes	4 (6.90%)	17 (1.88%)	27 (2.87%)	14 (6.11%)	<0.001
	No	54 (93.10%)	887 (98.12%)	915 (97.13%)	215 (93.89%)	
Heart disease	Yes	7 (12.07%)	75 (8.30%)	84 (8.92%)	35 (15.28%)	0.010
	No	51 (87.93%)	829 (91.70%)	858 (91.08%)	194 (84.72%)	
Stroke or cardiovascular diseases	Yes	7 (12.07%)	45 (4.98%)	41 (4.35%)	22 (9.61%)	<0.001
	No	51 (87.93%)	859 (95.02%)	901 (95.65%)	207 (90.39%)	
Lung diseases	Yes	8 (13.79%)	78 (8.63%)	100 (10.62%)	23 (10.04%)	0.365
	No	50 (86.21%)	826 (91.37%)	842 (89.38%)	206 (89.96%)	
Cataract	Yes	11 (18.97%)	92 (10.18%)	53 (5.63%)	24 (10.48%)	<0.001
	No	47 (81.03%)	812 (89.82%)	889 (94.37%)	205 (89.52%)	
Arthritis	Yes	9 (15.52%)	212 (23.45%)	205 (21.76%)	55 (24.02%)	0.444

	No	49 (84.48%)	692 (76.55%)	737 (78.24%)	174 (75.98%)	
**Physical function**						
ADL #	—	7.36±1.97	6.15±0.78	6.05±0.36	6.06±0.30	<0.001
IADL #	—	21.03±3.38	11.70±4.25	9.01±2.47	8.71±1.48	<0.001
**Sleep**						
Sleep quality	Very Good	9 (15.52%)	131 (14.49%)	147 (15.61%)	58 (25.33%)	<0.001
	Good	24 (41.38%)	449 (49.67%)	493 (52.34%)	118 (51.53%)	
	So so	15 (25.86%)	227 (25.11%)	217 (23.04%)	36 (15.72%)	
	Bad	10 (17.24%)	89 (9.85%)	76 (8.07%)	16 (6.99%)	
	Very bad	0 (0.00%)	8 (0.88%)	9 (0.96%)	1 (0.44%)	
Sleep duration #	—	8.57±2.66	7.87±2.22	7.59±1.91	7.57±1.77	<0.001
Smoking	Yes	5 (8.62%)	112 (12.39%)	342 (36.31%)	56 (24.45%)	<0.001
	No	53 (91.38%)	792 (87.61%)	600 (63.69%)	173 (75.55%)	
Drinking	Yes	11 (18.97%)	113 (12.50%)	319 (33.86%)	53 (23.14%)	<0.001
	No	47 (81.03%)	791 (87.50%)	623 (66.14%)	176 (76.86%)	
Exercise #	Yes	12 (20.69%)	256 (28.32%)	322 (34.18%)	175 (76.42%)	<0.001
	No	46 (79.31%)	648 (71.68%)	620 (65.82%)	54 (23.58%)	
**Lifestyles**						
Staple food	Rice	32 (55.17%)	563 (62.28%)	586 (62.21%)	119 (51.97%)	<0.001
	Corn (maize)	4 (6.90%)	28 (3.10%)	28 (2.97%)	10 (4.37%)	
	Wheat (noodles and bread etc.)	12 (20.69%)	194 (21.46%)	227 (24.10%)	52 (22.71%)	

	Rice and wheat	10 (17.24%)	119 (13.16%)	101 (10.72%)	48 (20.96%)	
Fruit	Almost everyday	14 (24.14%)	95 (10.51%)	114 (12.10%)	60 (26.20%)	<0.001
	Quite often	13 (22.41%)	254 (28.10%)	292 (31.00%)	64 (27.95%)	
	Occasionally	19 (32.76%)	340 (37.61%)	350 (37.15%)	71 (31.00%)	
	Rarely or never	12 (20.69%)	215 (23.78%)	186 (19.75%)	34 (14.85%)	
Vegetables	Almost everyday	40 (68.97%)	581 (64.27%)	622 (66.03%)	171 (74.67%)	0.054
	Quite often	13 (22.41%)	234 (25.88%)	257 (27.28%)	47 (20.52%)	
	Occasionally	4 (6.90%)	66 (7.30%)	51 (5.41%)	9 (3.93%)	
	Rarely or never	1 (1.72%)	23 (2.54%)	12 (1.27%)	2 (0.87%)	
What kind of grease do you mainly use for cooking	Animal fat	5 (8.62%)	155 (17.15%)	160 (16.99%)	18 (7.86%)	<0.001
	Plant oil	53 (91.38%)	749 (82.85%)	782 (83.01%)	211 (92.14%)	
Main flavor you have	No-salty	46 (79.31%)	759 (83.96%)	734 (77.92%)	183 (79.91%)	0.012
	Salty	12 (20.69%)	145 (16.04%)	208 (22.08%)	46 (20.09%)	
How often eat meat at present	Almost everyday	20 (34.48%)	257 (28.43%)	260 (27.60%)	77 (33.62%)	0.266
	Once a week	24 (41.38%)	336 (37.17%)	393 (41.72%)	80 (34.93%)	
	Once a month	7 (12.07%)	107 (11.84%)	117 (12.42%)	30 (13.10%)	
	Sometimes	4 (6.90%)	101 (11.17%)	89 (9.45%)	18 (7.86%)	
	Never	3 (5.17%)	103 (11.39%)	83 (8.81%)	24 (10.48%)	
How often eat fish at present	Almost everyday	7 (12.07%)	50 (5.53%)	71 (7.54%)	19 (8.30%)	<0.001
	Once a week	22 (37.93%)	264 (29.20%)	340 (36.09%)	96 (41.92%)	
	Once a month	12 (20.69%)	186 (20.58%)	193 (20.49%)	38 (16.59%)	

	Sometimes	8 (13.79%)	171 (18.92%)	166 (17.62%)	42 (18.34%)	
	Never	9 (15.52%)	233 (25.77%)	172 (18.26%)	34 (14.85%)	
How often eat eggs at present	Almost everyday	23 (39.66%)	303 (33.52%)	282 (29.94%)	104 (45.41%)	<0.001
	Once a week	23 (39.66%)	308 (34.07%)	352 (37.37%)	73 (31.88%)	
	Once a month	7 (12.07%)	108 (11.95%)	120 (12.74%)	17 (7.42%)	
	Sometimes	2 (3.45%)	94 (10.40%)	104 (11.04%)	17 (7.42%)	
	Never	3 (5.17%)	91 (10.07%)	84 (8.92%)	18 (7.86%)	
How often eat food made from beans at present #	Almost everyday	12 (20.69%)	139 (15.38%)	161 (17.09%)	60 (26.20%)	<0.001
	Once a week	31 (53.45%)	329 (36.39%)	348 (36.94%)	87 (37.99%)	
	Once a month	5 (8.62%)	142 (15.71%)	158 (16.77%)	24 (10.48%)	
	Sometimes	5 (8.62%)	163 (18.03%)	164 (17.41%)	36 (15.72%)	
	Never	5 (8.62%)	131 (14.49%)	111 (11.78%)	22 (9.61%)	
How often eat salt-preserved vegetables at present	Almost everyday	9 (15.52%)	202 (22.35%)	241 (25.58%)	44 (19.21%)	0.113
	Once a week	11 (18.97%)	144 (15.93%)	172 (18.26%)	39 (17.03%)	
	Once a month	2 (3.45%)	75 (8.30%)	79 (8.39%)	24 (10.48%)	
	Sometimes	9 (15.52%)	143 (15.82%)	155 (16.45%)	40 (17.47%)	
	Never	27 (46.55%)	340 (37.61%)	295 (31.32%)	82 (35.81%)	
How often eat sugar at present	Almost everyday	9 (15.52%)	152 (16.81%)	104 (11.04%)	39 (17.03%)	<0.001
	Once a week	16 (27.59%)	181 (20.02%)	190 (20.17%)	36 (15.72%)	
	Once a month	6 (10.34%)	97 (10.73%)	106 (11.25%)	20 (8.73%)	
	Sometimes	10 (17.24%)	190 (21.02%)	209 (22.19%)	36 (15.72%)	

	Never	17 (29.31%)	284 (31.42%)	333 (35.35%)	98 (42.79%)	
How often drink tea at present #	Almost everyday	11 (18.97%)	244 (26.99%)	388 (41.19%)	95 (41.48%)	<0.001
	Once a week	6 (10.34%)	47 (5.20%)	62 (6.58%)	9 (3.93%)	
	Once a month	1 (1.72%)	27 (2.99%)	17 (1.80%)	8 (3.49%)	
	Sometimes	6 (10.34%)	68 (7.52%)	63 (6.69%)	19 (8.30%)	
	Never	34 (58.62%)	518 (57.30%)	412 (43.74%)	98 (42.79%)	
How often eat garlic at present	Almost everyday	8 (13.79%)	186 (20.58%)	238 (25.27%)	53 (23.14%)	<0.001
	Once a week	18 (31.03%)	208 (23.01%)	225 (23.89%)	55 (24.02%)	
	Once a month	4 (6.90%)	103 (11.39%)	116 (12.31%)	15 (6.55%)	
	Sometimes	16 (27.59%)	185 (20.46%)	199 (21.13%)	51 (22.27%)	
	Never	12 (20.69%)	222 (24.56%)	164 (17.41%)	55 (24.02%)	
How often eat milk products at present	Almost everyday	21 (36.21%)	140 (15.49%)	144 (15.29%)	64 (27.95%)	<0.001
	Once a week	4 (6.90%)	88 (9.73%)	85 (9.02%)	32 (13.97%)	
	Once a month	4 (6.90%)	66 (7.30%)	67 (7.11%)	11 (4.80%)	
	Sometimes	8 (13.79%)	124 (13.72%)	131 (13.91%)	32 (13.97%)	
	Never	21 (36.21%)	486 (53.76%)	515 (54.67%)	90 (39.30%)	
How often eat nut products at present	Almost everyday	1 (1.72%)	19 (2.10%)	49 (5.20%)	15 (6.55%)	<0.001
	Once a week	8 (13.79%)	68 (7.52%)	116 (12.31%)	33 (14.41%)	
	Once a month	7 (12.07%)	60 (6.64%)	106 (11.25%)	20 (8.73%)	
	Sometimes	10 (17.24%)	177 (19.58%)	247 (26.22%)	55 (24.02%)	
	Never	32 (55.17%)	580 (64.16%)	424 (45.01%)	106 (46.29%)	

How often eat mushroom or algae at present	Almost everyday	0 (0.00%)	14 (1.55%)	16 (1.70%)	7 (3.06%)	<0.001
	Once a week	9 (15.52%)	78 (8.63%)	108 (11.46%)	52 (22.71%)	
	Once a month	9 (15.52%)	101 (11.17%)	136 (14.44%)	26 (11.35%)	
	Sometimes	20 (34.48%)	264 (29.20%)	280 (29.72%)	64 (27.95%)	
	Never	20 (34.48%)	447 (49.45%)	402 (42.68%)	80 (34.93%)	
How often eat vitamins (a/c/e) at present	Almost everyday	4 (6.90%)	55 (6.08%)	41 (4.35%)	36 (15.72%)	<0.001
	Once a week	2 (3.45%)	30 (3.32%)	26 (2.76%)	7 (3.06%)	
	Once a month	4 (6.90%)	18 (1.99%)	26 (2.76%)	7 (3.06%)	
	Sometimes	13 (22.41%)	106 (11.73%)	120 (12.74%)	21 (9.17%)	
	Never	35 (60.34%)	695 (76.88%)	729 (77.39%)	158 (69.00%)	
How often eat medicinal plants at present	Almost everyday	0 (0.00%)	12 (1.33%)	27 (2.87%)	7 (3.06%)	0.080
	Once a week	2 (3.45%)	15 (1.66%)	21 (2.23%)	11 (4.80%)	
	Once a month	2 (3.45%)	23 (2.54%)	26 (2.76%)	7 (3.06%)	
	Sometimes	9 (15.52%)	97 (10.73%)	87 (9.24%)	20 (8.73%)	
	Never	45 (77.59%)	757 (83.74%)	781 (82.91%)	184 (80.35%)	
**Mental health**						
Self-reported quality of life	Very good	10 (17.24%)	138 (15.27%)	130 (13.80%)	62 (27.07%)	<0.001
	Good	25 (43.10%)	427 (47.23%)	414 (43.95%)	100 (43.67%)	
	So so	14 (24.14%)	275 (30.42%)	352 (37.37%)	62 (27.07%)	
	Bad	8 (13.79%)	57 (6.31%)	41 (4.35%)	4 (1.75%)	
	Very bad	1 (1.72%)	7 (0.77%)	5 (0.53%)	1 (0.44%)	

Self-reported health	Very good	6 (10.34%)	116 (12.83%)	141 (14.97%)	34 (14.85%)	<0.001
	Good	17 (29.31%)	372 (41.15%)	376 (39.92%)	102 (44.54%)	
	So so	18 (31.03%)	296 (32.74%)	299 (31.74%)	63 (27.51%)	
	Bad	13 (22.41%)	110 (12.17%)	118 (12.53%)	28 (12.23%)	
	Very bad	4 (6.90%)	10 (1.11%)	8 (0.85%)	2 (0.87%)	
Feeling health change	Much better	2 (3.45%)	17 (1.88%)	21 (2.23%)	11 (4.80%)	<0.001
	A little better	7 (12.07%)	78 (8.63%)	83 (8.81%)	21 (9.17%)	
	No change	19 (32.76%)	495 (54.76%)	566 (60.08%)	121 (52.84%)	
	A little worse	23 (39.66%)	281 (31.08%)	243 (25.80%)	70 (30.57%)	
	Much worse	7 (12.07%)	33 (3.65%)	29 (3.08%)	6 (2.62%)	
Being positive	Always	7 (12.07%)	105 (11.62%)	133 (14.12%)	63 (27.51%)	<0.001
	Often	45 (77.59%)	606 (67.04%)	631 (66.99%)	136 (59.39%)	
	Sometimes	5 (8.62%)	148 (16.37%)	139 (14.76%)	20 (8.73%)	
	Seldom	1 (1.72%)	41 (4.54%)	36 (3.82%)	8 (3.49%)	
	Never	0 (0.00%)	4 (0.44%)	3 (0.32%)	2 (0.87%)	
Hygiene #	Always	11 (18.97%)	116 (12.83%)	125 (13.27%)	74 (32.31%)	<0.001
	Often	35 (60.34%)	574 (63.50%)	561 (59.55%)	127 (55.46%)	
	Sometimes	12 (20.69%)	202 (22.35%)	246 (26.11%)	26 (11.35%)	
	Seldom	0 (0.00%)	11 (1.22%)	10 (1.06%)	2 (0.87%)	
	Never	0 (0.00%)	1 (0.11%)	0 (0.00%)	0 (0.00%)	
Anxiety	Always	0 (0.00%)	11 (1.22%)	8 (0.85%)	9 (3.93%)	<0.001

	Often	5 (8.62%)	41 (4.54%)	29 (3.08%)	6 (2.62%)	
	Sometimes	12 (20.69%)	189 (20.91%)	174 (18.47%)	26 (11.35%)	
	Seldom	22 (37.93%)	290 (32.08%)	305 (32.38%)	72 (31.44%)	
	Never	19 (32.76%)	373 (41.26%)	426 (45.22%)	116 (50.66%)	
Loneliness #	Always	2 (3.45%)	18 (1.99%)	6 (0.64%)	6 (2.62%)	<0.001
	Often	1 (1.72%)	58 (6.42%)	35 (3.72%)	8 (3.49%)	
	Sometimes	13 (22.41%)	196 (21.68%)	166 (17.62%)	25 (10.92%)	
	Seldom	20 (34.48%)	267 (29.54%)	296 (31.42%)	60 (26.20%)	
	Never	22 (37.93%)	365 (40.38%)	439 (46.60%)	130 (56.77%)	
Decision-making #	Always	16 (27.59%)	295 (32.63%)	371 (39.38%)	117 (51.09%)	<0.001
	Often	21 (36.21%)	198 (21.90%)	305 (32.38%)	57 (24.89%)	
	Sometimes	15 (25.86%)	251 (27.77%)	177 (18.79%)	43 (18.78%)	
	Seldom	6 (10.34%)	119 (13.16%)	62 (6.58%)	10 (4.37%)	
	Never	0 (0.00%)	41 (4.54%)	27 (2.87%)	2 (0.87%)	
Feeling useless	Always	5 (8.62%)	65 (7.19%)	48 (5.10%)	9 (3.93%)	<0.001
	Often	10 (17.24%)	182 (20.13%)	130 (13.80%)	24 (10.48%)	
	Sometimes	22 (37.93%)	339 (37.50%)	318 (33.76%)	72 (31.44%)	
	Seldom	11 (18.97%)	164 (18.14%)	233 (24.73%)	51 (22.27%)	
	Never	10 (17.24%)	154 (17.04%)	213 (22.61%)	73 (31.88%)	
Feeling happy	Always	7 (12.07%)	73 (8.08%)	72 (7.64%)	18 (7.86%)	0.024
	Often	9 (15.52%)	175 (19.36%)	177 (18.79%)	48 (20.96%)	

	Sometimes	14 (24.14%)	287 (31.75%)	366 (38.85%)	94 (41.05%)	
	Seldom	21 (36.21%)	260 (28.76%)	240 (25.48%)	50 (21.83%)	
	Never	7 (12.07%)	109 (12.06%)	87 (9.24%)	19 (8.30%)	
**Leisure activity**						
Garden work #	Almost everyday	1 (1.72%)	85 (9.40%)	125 (13.27%)	54 (23.58%)	<0.001
	Once a week	0 (0.00%)	19 (2.10%)	24 (2.55%)	10 (4.37%)	
	Once a month	1 (1.72%)	17 (1.88%)	13 (1.38%)	5 (2.18%)	
	Sometimes	1 (1.72%)	20 (2.21%)	24 (2.55%)	10 (4.37%)	
	Never	55 (94.83%)	763 (84.40%)	756 (80.25%)	150 (65.50%)	
Reading	Almost everyday	5 (8.62%)	38 (4.20%)	144 (15.29%)	73 (31.88%)	<0.001
	Once a week	2 (3.45%)	24 (2.65%)	49 (5.20%)	17 (7.42%)	
	Once a month	2 (3.45%)	12 (1.33%)	38 (4.03%)	13 (5.68%)	
	Sometimes	1 (1.72%)	24 (2.65%)	52 (5.52%)	15 (6.55%)	
	Never	48 (82.76%)	806 (89.16%)	659 (69.96%)	111 (48.47%)	
Raising pets	Almost everyday	3 (5.17%)	197 (21.79%)	313 (33.23%)	54 (23.58%)	<0.001
	Once a week	1 (1.72%)	22 (2.43%)	42 (4.46%)	1 (0.44%)	
	Once a month	0 (0.00%)	15 (1.66%)	15 (1.59%)	3 (1.31%)	
	Sometimes	1 (1.72%)	25 (2.77%)	27 (2.87%)	8 (3.49%)	
	Never	53 (91.38%)	645 (71.35%)	545 (57.86%)	163 (71.18%)	
Playing cards or mah-jongg	Almost everyday	1 (1.72%)	49 (5.42%)	89 (9.45%)	40 (17.47%)	<0.001
	Once a week	3 (5.17%)	42 (4.65%)	58 (6.16%)	12 (5.24%)	

	Once a month	0 (0.00%)	21 (2.32%)	34 (3.61%)	8 (3.49%)	
	Sometimes	2 (3.45%)	27 (2.99%)	77 (8.17%)	15 (6.55%)	
	Never	52 (89.66%)	765 (84.62%)	684 (72.61%)	154 (67.25%)	
Watch TV or listen to radio #	Almost everyday	34 (58.62%)	437 (48.34%)	677 (71.87%)	220 (96.07%)	<0.001
	Once a week	3 (5.17%)	113 (12.50%)	98 (10.40%)	9 (3.93%)	
	Once a month	2 (3.45%)	45 (4.98%)	46 (4.88%)	0 (0.00%)	
	Sometimes	2 (3.45%)	51 (5.64%)	38 (4.03%)	0 (0.00%)	
	Never	17 (29.31%)	258 (28.54%)	83 (8.81%)	0 (0.00%)	
Social activities	Almost everyday	1 (1.72%)	16 (1.77%)	34 (3.61%)	28 (12.23%)	<0.001
	Once a week	0 (0.00%)	10 (1.11%)	30 (3.18%)	10 (4.37%)	
	Once a month	0 (0.00%)	22 (2.43%)	45 (4.78%)	15 (6.55%)	
	Sometimes	1 (1.72%)	33 (3.65%)	75 (7.96%)	20 (8.73%)	
	Never	56 (96.55%)	823 (91.04%)	758 (80.47%)	156 (68.12%)	
**Social support**						
Are personal care services available in your community	Yes	1 (1.72%)	17 (1.88%)	28 (2.97%)	11 (4.80%)	0.082
	No	57 (98.28%)	887 (98.12%)	914 (97.03%)	218 (95.20%)	
Are home visit services available in your community	Yes	4 (6.90%)	47 (5.20%)	68 (7.22%)	25 (10.92%)	0.018
	No	54 (93.10%)	857 (94.80%)	874 (92.78%)	204 (89.08%)	
Are psychological consulting services available in your community	Yes	5 (8.62%)	37 (4.09%)	40 (4.25%)	18 (7.86%)	0.040
	No	53 (91.38%)	867 (95.91%)	902 (95.75%)	211 (92.14%)	
	Yes	2 (3.45%)	30 (3.32%)	40 (4.25%)	16 (6.99%)	0.097

Are daily shopping services available in your community	No	56 (96.55%)	874 (96.68%)	902 (95.75%)	213 (93.01%)	
Are social and recreation services available in your community	Yes	7 (12.07%)	59 (6.53%)	100 (10.62%)	36 (15.72%)	<0.001
	No	51 (87.93%)	845 (93.47%)	842 (89.38%)	193 (84.28%)	
Are legal aid services available in your community	Yes	2 (3.45%)	36 (3.98%)	63 (6.69%)	19 (8.30%)	0.017
	No	56 (96.55%)	868 (96.02%)	879 (93.31%)	210 (91.70%)	
Are healthcare education services available in your community	Yes	7 (12.07%)	49 (5.42%)	76 (8.07%)	26 (11.35%)	<0.001
	No	51 (87.93%)	855 (94.58%)	866 (91.93%)	203 (88.65%)	
Are neighborhood-relation services available in your community	Yes	11 (18.97%)	131 (14.49%)	145 (15.39%)	55 (24.02%)	<0.001
	No	47 (81.03%)	773 (85.51%)	797 (84.61%)	174 (75.98%)	

#The 20 features used in the clustering analysis. Abbreviations: ADL, activities of daily living; IADL, instrumental activities of daily living; BMI, body mass index;
